# Lymphatics and Lymphangiogenesis in the Eye

**DOI:** 10.1155/2012/783163

**Published:** 2012-03-05

**Authors:** Shintaro Nakao, Ali Hafezi-Moghadam, Tatsuro Ishibashi

**Affiliations:** ^1^Department of Ophthalmology, Graduate School of Medical Sciences, Kyushu University, 3-1-1 Maidashi, Higashi-Ku, Fukuoka 812-8582, Japan; ^2^Functional and Molecular Imaging Center and Department of Radiology, Brigham and Women*ʼ*s Hospital, Harvard Medical School, Boston, MA 02115, USA

## Abstract

Lymphatic is a prerequisite for the maintenance of tissue fluid balance and immunity in the body. A body of evidence also shows that lymphangiogenesis plays important roles in the pathogenesis of diseases such as tumor metastasis and inflammation. The eye was thought to lack lymphatic vessels except for the conjunctiva; however, advances in the field, including the identification of lymphatic endothelial markers (e.g., LYVE-1 or podoplanin) and lymphangiogenic factors (e.g., VEGF-C), have revealed the exsitence and possible roles of lymphatics and lymphangiogenesis in the eye. Recent studies have shown that corneal limbus, ciliary body, lacrimal gland, orbital meninges, and extraocular muscles contain lymphatic vessels and that the choroid might have a lymphatic-like system. There is no known lymphatic outflow from the eye. However, several lymphatic channels including uveolymphatic pathway might serve the ocular fluid homeostasis. Furthermore, lymphangiogenesis plays important roles in pathological conditions in the eye including corneal transplant rejection and ocular tumor progression. Yet, the role of lymphangiogenesis in most eye diseases, especially inflammatory disease or edema, remains unknown. A better understanding of lymphatic and lymphangiogenesis in the eye will open new therapeutic opportunities to prevent vision loss in ocular diseases.

## 1. Introduction

The lymphatic system in human was first described by Gasper Aselli in 1627 in a paper “De Lacteibus sive Lacteis Venis,” Quarto Vasorum Mesarai corum Genere novo invento. Now, it is well known that the lymphatics remove interstitial fluid and macromolecules, including proteins, and transport them to lymph nodes before entering the blood circulation. From the lymphatic capillaries, the lymph is transported via precollectors to collecting lymphatic vessels and is returned through the lymphaticovenous junctions between the thoracic or lymphatic duct and the subclavian veins to the blood circulation [1]. Another role of the lymphatics is to carry immune cells to the lymph nodes and to control the immunity in health and disease.

Although the presence of lymphatics was long known through histology [2], systematic lymphatic research started later than blood vessels because of lack of specific markers. Recent identification of lymphatic endothelial markers facilitated lymphatic research [3, 4]. Furthermore, finding of lymphangiogenic factors reveals various mechanisms of lymphangiogenesis in health and disease. For instance, lymphangiogenesis plays critical roles in various disorders, including cancer metastasis and inflammation [5, 6].

In the past two decades, lymphatics and lymphangiogenesis in the eye and the phenotypes in the various ocular diseases have been investigated. Histological studies show the location and existence of lymphatics in the eye [7]. These studies have revealed that lymphatics contribute to the ocular homeostasis and that ocular lymphangiogenesis may play important roles in eye disorders. This paper reviews current knowledge on lymphatics and lymphangiogenesis in the eye and discusses the possibility of lymphatic-targeting therapy.

## 2. VEGF/VEGFR System in Lymphangiogenesis

VEGF family is important for vasculogenesis, angiogenesis, and lymphangiogenesis [8]. The mammalian VEGF family presently contains five members: VEGF-A, placenta growth factor (PlGF), VEGF-B, VEGF-C, and VEGF-D. VEGF-A has important roles in mammalian vascular development and in diseases involving abnormal growth of blood vessels. Recent clinical studies have demonstrated the significance of VEGF-A in ocular neovascularization (e.g., diabetic retinopathy and aged-macular degeneration) with use of VEGF-A neutralizing antibodies [9]. VEGF-C and VEGF-D are main lymphangiogenic factors in both physiological and pathological conditions. The VEGF receptor family contains three members: VEGFR-1 (Flt-1), VEGFR-2 (KDR/Flk-1), and VEGFR-3 (Flt-4). VEGF-A binds and activates two tyrosine kinase receptors: VEGFR-1 and VEGFR-2 [8]. VEGF-A does not show any appreciable binding affinity to VEGFR-3. VEGFR-3 is a ligand for VEGF-C and VEGF-D. Mature form of VEGF-C and human VEGF-D are known to bind and activate VEGFR-2 [10, 11]. Various studies clarify the involvement of VEGF-C and -D/VEGFR-3 system in cancer lymphatic invasion and lymph node metastasis [12, 13]. Furthermore VEGF-C and -D/VEGFR-3 signaling is involved in inflammatory diseases and organ transplantation [6, 14, 15].

## 3. The Other Lymphangiogenesis-Related Factors

 Lymphangiogenic factors include not only VEGF family but also the other growth factors and cytokines such as insulin-like growth factors (IGFs), hepatocyte growth factor (HGF), fibroblast growth factors (FGFs), and interleukins (ILs). These growth factors and cytokines have been well known to be angiogenic. Some of these factors can cause lymphangiogenesis directly and some can induce lymphangiogenesis via VEGF family indirectly. These findings are shown with assays in cornea.

## 4. Corneal Avascularity and Its Alymphatic Characteristics

**Figure 1:**
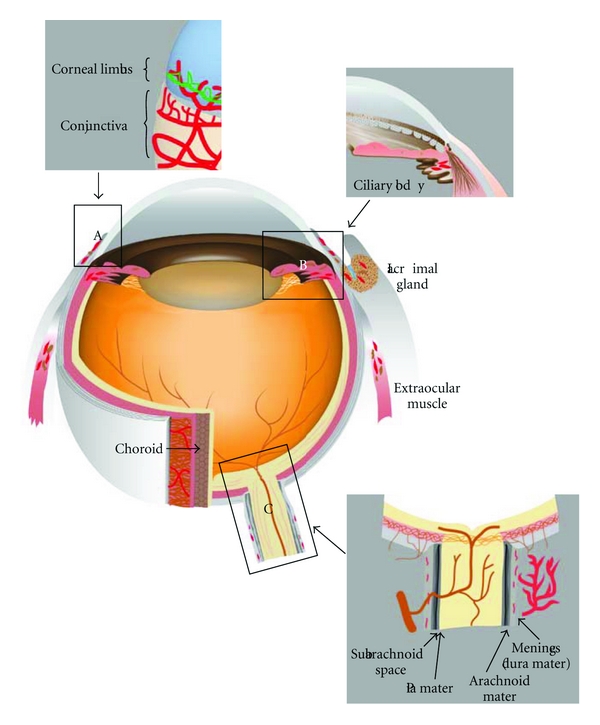
Distribution of lymphatics in the eye. Immunological staining with lymphatic specific markers as well as histological examinations has revealed the distribution of lymphatic vessels in the eye. Conjunctiva is well known to possess lymphatics. Cornea limbus, ciliary body, lacrimal gland, orbital meninges and extraocular muscle also contain lymphatic vessels, and choroid might have lymphatic-like system. Cornea, retina, and optic nerve do not show lymphatics. Intraocular lymphatic can be observed only in the ciliary body. (A), (B), and (C) show the details of corneal limbal area, angulus iridocornealis, and optic nerve, respectively. Red indicates lymphatic vessels.

 The normal cornea, but not the conjunctiva, is devoid of lymphatic vessels as well as blood vessels (Figure 1). The alymphatic mechanism was unknown until recently a study showed that a soluble VEGFR-2 form is secreted by corneal epithelial cells selectively suppressing the physiologic growth of lymphatics [16]. This finding is the first identification of a specific lymphangiogenesis inhibitor.

## 5. Corneal Lymphangiogenesis Assay

**Figure 2:**
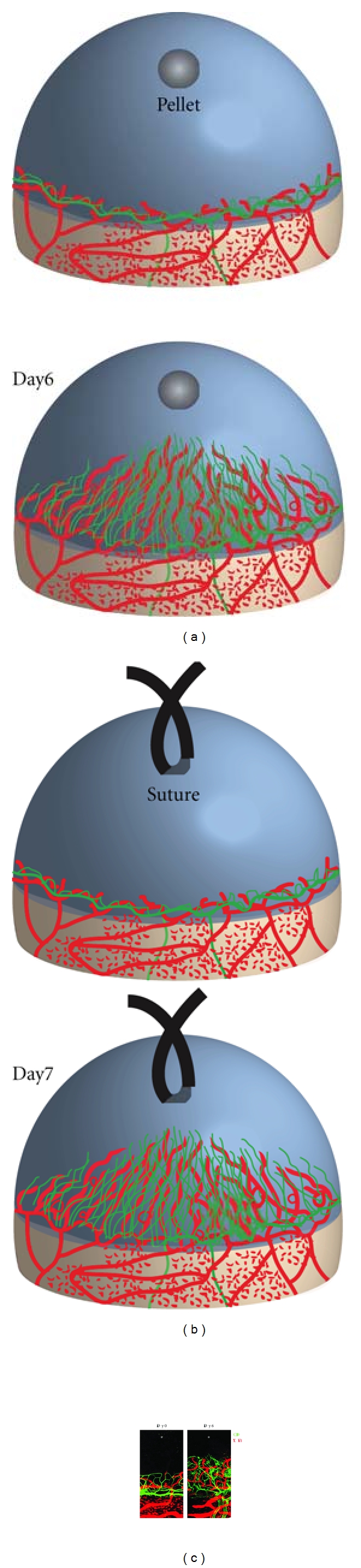
Mouse cornea lymphangiogenesis model. No lymphatic vessels exist in the normal mammalian cornea. Implantation of growth factor or cytokine into a surgically created micropocket in the mouse cornea stroma (a) or corneal injury by suture (b) induces lymphangiogenic response. Corneal lymphangiogenesis as well as angiogenesis can be examined 6 or 7 days after pellet implantation or suture, respectively (a) and (b). Double staining of corneal flat mounts for lymphangiogenic (LYVE-1, red) endothelium and angiogenic (CD31, green) with immunohistochemistry (c).

 Because of its avascularity, the cornea is widely used to investigate lymphangiogenesis. One of the most reliable methods to examine lymphangiogenesis is corneal inflammation model by suture or alkali burn (NaOH solution) [17, 18] (Figure 2). Another authentic method is cornea micropocket assay, which has been used for estimation of angiogenesis since the 1970s [19, 20] (Figure 2). In both models, lymphangiogenesis occurs from preexisting limbal lymphatics. The cornea micropocket model has revealed that most angiogenic factors also induce lymphangiogenesis (Table 1). This might indicate that the mechanism of lymphangiogenesis is partially similar with angiogenesis. IGF-1 and IGF-2, which significantly stimulates proliferation and migration of lymphatic endothelial cells, can induce corneal lymphangiogenesis. IGF-1-induced lymphangiogenesis is not mediated by VEGFR-3 signaling [21]. A potent angiogenic factor, VEGF-A, is also shown to be a lymphangiogenic factor in mouse cornea [22]. In VEGF-A-induced lymphangiogenesis, there are both mechanisms: VEGFR-3-dependent and VEGFR-3-independent. VEGF-A can induce the proliferation of lymphatic endothelial cells directly, which is not mediated by VEGFR-3 [23]. This lymphangiogenesis is also mediated by macrophage-derived VEGF-C with the inflammatory suture model [24]. The balance between direct and indirect effect in VEGF-A-induced lymphangiogenesis may depend on the situations. VEGF-C, a potent lymphangiogenic factor, was confirmed to induce corneal lymphangiogenesis [25]. VEGF-C156S, which is a specific ligand for VEGFR-3, also causes lymphangiogenesis as well as angiogenesis in the cornea [26]. This VEGFR-3-mediated lymphangiogenesis could be induced by direct effect for lymphatic endothelium as well as macrophage recruitment. Platelet-derived growth factor (PDGF) causes corneal lymphangiogenesis via direct stimulation of lymphatic endothelium [27]. HGF also causes corneal lymphangiogenesis that can be blocked by VEGFR-3 inhibition partially [28]. FGF-2, which is a well-known potent angiogenic factor, could cause lymphangiogenesis with corneal micropocket assay [29]. The FGF-2-induced lymphangiogenesis was blocked by VEGFR-3 inhibition [29]. These investigations suggest that each GF (growth factor) has different dependency on VEGFR-3 signaling in lymphangiogenesis. Some GFs cause proliferation or migration of lymphatic endothelial cells directly, whereas some GFs upregulate VEGF-C/-D to activate VEGFR-3 in lymphangiogenesis. Interestingly, the corneal micropocket assay reveals that low dose of FGF-2 causes selective lymphangiogenesis [30]. This observation provides the evidence that lymphatic growth is possible without angiogenesis. However, the detailed mechanism of FGF-2-mediated lymphangiogenesis has been enigmatic. A recent examination using mouse cornea introduced physiological expression of lymphatics without the presence of blood vessels, which is an indication that angiogenesis and lymphangiogenesis might occur independently [25]. In the study, FGF-2-deficient mice show significantly less preexisting lymphatic sprouts without having an effect on angiogenesis in the cornea compared to their wild-type counterparts [25]. Consequently, this suggests that lymph- and angiogenesis might occur independently.

## 6. Genetic Heterogeneity of Lymphangiogenesis

**Figure 3:**
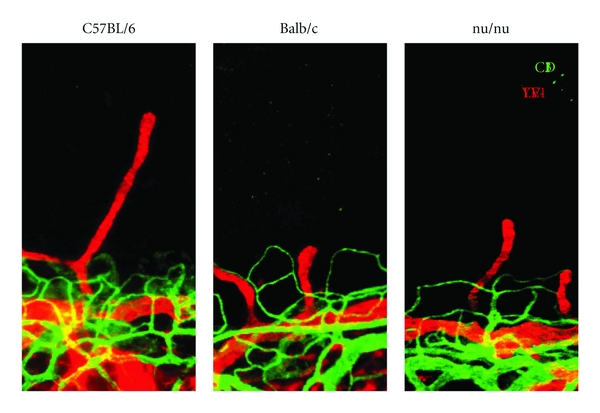
Strain-dependent limbal lymphatics. Genetic background significantly affects corneal preexisting limbal lymphatics. The area of preexisting lymphatics in C57BL/6 mice is significantly larger than that in Balb/c mice. *nu/nu* mice show the intermediate phenotype. Preexisting limbal lymphatics may be heterogenic in patients, causing differences in corneal transplant rejection or keratitis. *nu/nu* mice develop normal lymphatics, suggesting that T cells may not be important for lymphatic development.

 Recently two different groups have reported on genetic heterogeneity of corneal lymphangiogenesis in different mouse strains with the corneal suture model and the corneal micropocket model independently [25, 34] (Figure 3). This suggests that heterogeneity of corneal lymphatics shows different inflammatory reactions in patients. In the comparative analysis of lymphatics with various strains, *nu/nu* mice, which have a greatly reduced number of T cells, showed similar lymphatic development and GF-induced lymphangiogenesis with the other strains, suggesting that T cells might be unnecessary for lymphatic development as well as GF-induced lymphangiogenesis in the cornea [25] (Figure 3). Inflammation model by suture has also revealed various insights of lymphangiogenesis. After corneal inflammation, pathologic corneal lymphangiogenesis can regress earlier than angiogenic vessels [17].

## 7. The Interplay between Angiogenesis and Lymphangiogenesis

 As described above, lymphangiogenesis and angiogenesis occur in concert [6]. However, how blood and lymphatic vessels regulate each other has been unknown. Recently, it was reported that angiogenic vessels delay lymphangiogenesis using corneal micropocket assay [31]. In response to VEGF-A, corneal lymphatics grow with a delay compared to blood vessels. Higher concentrations of VEGF-A are needed for lymphangiogenesis than for angiogenesis. The poised temporal and spatial association of angio- and lymphangiogenesis indicates interdependencies between blood and lymphatic vessels. Proteolytically processed VEGF-C binds to and activates VEGFR-2. Upregulated VEGFR-2 in angiogenic tips could trap VEGF-C, and the trapped VEGF-C could not reach lymphangiogenesis due to the distance apart from the lymphatic vessels [31]. This VEGF-C/VEGFR-2 interaction might regulate the relation between angio- and lymphangiogenesis. Delayed lymphangiogenesis might allow immune cells additional time at the inflammatory sites because immune cells originate from angiogenic vessels and drained through lymphangiogenic vessels [35].

## 8. Macrophages in Lymphangiogenesis

Macrophages have been well investigated for their role in neovascularization in various ocular diseases including ker atitis [36, 37], retinal angiogenesis [38, 39], and choroidal neovascularization [40, 41]. Macrophages contribute to corneal lymphangiogenesis in two different ways. Maruyama et al. showed that CD11b(+) macrophages infiltrate the inflammatory cornea and transdifferentiate into lymphatic endothelium that contributes to lymphangiogenesis [42]. Another role of macrophages is to provide lymphangiogenic factors. During corneal inflammation, infiltrating macrophage activates the NF-*κ*B signaling and secretes the downstream cytokines (e.g., VEGF-A, -C, and -D) to induce corneal lymphangiogenesis [32]. NF-*κ*B inhibition could block corneal lymphangiogenesis as well as the angiogenesis. A recent paper showed a mechanism for macrophages to infiltrate into the sites of corneal lymphangiogenesis. Vascular adhesion protein-1 (VAP-1) is an endothelial glycoprotein that regulates leukocyte transmigration [43, 44]. VAP-1 inhibition blocks inflammatory corneal lymphangiogenesis by reducing macrophage infiltration [33]. Macrophage polarization (M1 classical versus M2 alternatively activated macrophages) was recently discovered to regulate various inflammatory diseases [45]. The number of M2 marker(+) macrophages in inflammatory corneas of VAP-1-inhibitor-treated mice was significantly lower than in vehicle-treated mice [33]. Thus, M2 macrophages might play an important role in corneal lymphangiogenesis. However, further investigation will be necessary to discern the role of macrophage polarization in lymphangiogenesis. VAP-1 may become a therapeutic target for various lymphangiogenesis-related ocular diseases. Furthermore, a recent report showed that the antiangiogenic factor Thrombospondin-1 (TSP-1) is also an endogenous antilymphangiogenic factor [46]. TSP-1 can suppress macrophage-derived VEGF-C and VEGF-D by ligating CD36 on the cells. As a result, TSP-1 can become a therapeutic molecule for corneal lymphangiogenesis.

## 9. Lymphangiogenesis in Corneal Disorders

 Human cornea lacks lymphatic vessels during the development [47]. Vascularization in cornea disturbs visual acuity, whereas corneal lymphangiogenesis cannot. However, lymphangiogenesis in the cornea can modulate corneal immunity or inflammation. Increasing studies on lymphatic and lymphangiogenesis, have shown that lymphatic vessels play an important role for various corneal disorders (Table 2). Herpes simplex virus-1 (HSV-1) infection in the cornea is a leading cause of blindness. Corneal HSV-1 infection induces lymphangiogenesis, and the corneal lymphatics persist past the resolution of infection (Table 2). HSV-1-elicited lymphangiogenesis was reported to be strictly dependent on VEGF-A/VEGFR-2 signaling but not on VEGFR-3 ligands [48]. A recent study also showed that dry eye, a low-grade corneal inflammatory disorder, induces lymphangiogenesis by the upregulation of VEGFs and VEGFRs and CD11b(+) macrophage recruitment. Interestingly, corneal lymphangiogenesis in dry eye does not accompany angiogenesis (Table 2). However, the mechanism is not fully appreciated, and further investigation must include the estimation that lymphangiogenesis can be a therapeutic target for dry eye disease [49].

## 10. The Role of Lymphangiogenesis in Corneal Graft Rejection

**Figure 4:**
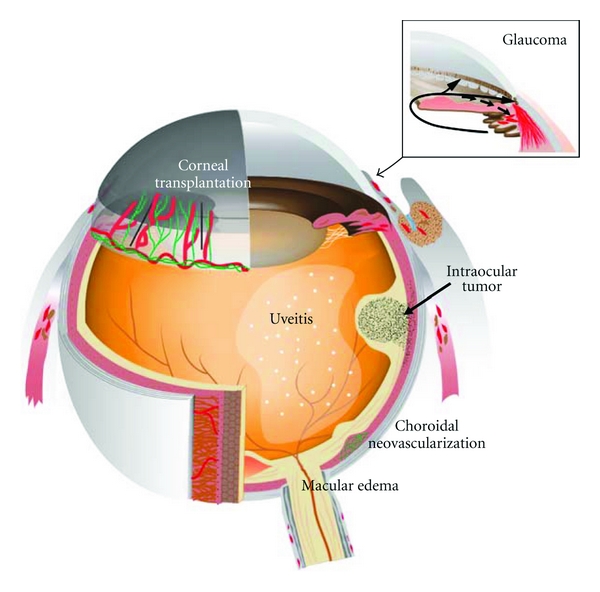
Lymphangiogenesis as a possible therapeutic target for the eye diseases. Corneal transplant, glaucoma, and intraocular tumors were suggested to be related to lymphatics or lymphangiogenesis in the pathogenesis. If choroid has lymphatic function, lymphatics or lymphangiogenesis may be important for the pathology of uveitis, choroidal neovascularization, or macular edema because of the vital role of lymphatics in inflammation or tissue edema. Red: lymphatic vessels. Green: blood vessels.

 The critical role of lymph nodes in corneal alloimmunization and graft rejection has been well investigated [54]. VEGFR-3 blockade suppressed corneal antigen-presenting cell trafficking to the lymph node and delayed the rejection of corneal transplanted graft rejection [55]. Lymphatic vessels, but not angiogenic vessels, could be important for immune rejection after corneal transplantation [50] (Table 2). Lymphatics and lymphangiogenesis-related factor would be a therapeutic target for corneal graft rejection (Figure 4).

## 11. Conjunctival Lymphatics in Glaucoma Surgery

Conjunctiva is the most lymphatic-developed tissue in the eye. A surgeon incidentally fills anesthetic solution into the lymphatics of a patient's conjunctiva during an operation. The doctor routinely visualizes conjunctival lymphatic vessels with a dye to decide where to make scleral filter to lower intraocular pressure (IOP) in glaucoma patients. Interestingly, he suggests that a healthy lymphatic system in the conjunctiva may decide the outcome of lowering IOP surgery and alerts that mitomycin or cauterization can cause damage to the lymphatic structures [56].

## 12. Lymphangiogenesis in Conjunctivitis

Various studies with LYVE-1 or podoplanin antibody confirmed that lymphatic vessels exist in conjunctiva in various species. Corneal lymphangiogenesis is sprouted from limbal lymphatics that connect to conjunctival lymphatic [57]. The removal of conjunctiva could not affect GF-induced corneal lymphangiogenesis, suggesting that the conjunctiva, including its LYVE-1(+) lymphatics and cells, may not be necessary for corneal lymphangiogenesis [25]. However, lymphatics might play an important role to heal conjunctivitis or conjunctival chemosis, or probably corneal edema. Furthermore, a group reported that injected tracers in the anterior chamber or the vitreous utilize conjunctival lymphatics to reach the lymph nodes [58, 59]. These data alert that intraocular drug injection affects the immunity.

## 13. The Possibility of Lymphatic-Targeting Therapy in Retinal Disorders

 Retina is part of the central nervous system (CNS) that is vascularized and has been thought not to have lymphatics as well as other parts of the CNS like the brain [60]. In addition to severe vision loss, macular edema is commonly associated with many retinal diseases including diabetic macular edema and retinal vein occlusion [61]. It is believed to be caused by hyperpermeability of the retinal vessels and/or decreased efflux of fluid across the retinal pigment epithelium, which can be induced by outer/inner blood retinal barrier dysfunction. In the past decade, there were advances in therapy for macular edema. Administration of steroid or VEGF neutralizing antibodies or vitrectomy can reduce macular edema significantly in patients [62]. However, these treatments may cause adverse effects including an increased incidence of IOP elevation or tractional retinal detachment [63, 64]. These pharmacological mechanisms to reduce macular edema must be caused by the blockade of leakage from retinal vessels [65–67]. However, the mechanism to absorb leaked interstitial fluid in macular edema is unclear. A recent paper suggested that drainage from the vitreous might exist via conjunctival lymphatics [59]. Furthermore, as described below, choroid might have lymphatic-like system. It has been reported that the dysfunction of lymphatic vessels causes lymphedema or tissue edema in various diseases and that lymphatic normalization or newly lymphatic vessels reduce tissue edema in the skin [68]. Further investigations of the lymphatic role of the posterior eye segment may reveal novel ways to manage macular edema (Figure 4).

 Recently podoplanin is shown to be expressed in retinal pigment epithelium (RPE) [69]. Podoplanin depletion with siRNA reduces cell aggregation, proliferation, and the tight junction. RPE regulates outer blood-retinal barrier, which is broken down in various retinal diseases. Further research is required to reveal how podoplanin in RPE contributes to the pathogenesis of retinal disorders.

## 14. Choroidal Lymphatics: A Controversial Point

 The choroid, which forms the uvea with the ciliary body and the iris, is one of the most highly vascularized tissue in the body. The choroid provides oxygen and nourishment to the outer layers of the retina. Before the discoveries of lymphatic specific markers, various histological examinations of animal choroid have shown the existence of lymphatic vessels in the choroid (Figure 1). In 1997, a paper on the avian eye proposed that the lacunae of the choroid represented a system with short lymphatic vessels that reached the choriocapillaris [70]. The authors proposed that the system might drain intraocular fluids directly into the eye venous system. In 1998, W. krebs ad I. P. krebs showed evidence of choroidal lymphatic vessels in the monkey by using electron microscopy [71]. In this paper, their observation with lymphatic specific features, including the lack of a continuous external basement lamina and the presence of anchoring filaments, supported their conclusion. Another study examined whether lymphatic vessels existed in monkey choroid [72]. This electron microscopic study observed the lymphatic sinus-like structures in the outer choroid. The lymphatic sinus-like structures were lined with fibroblast-like cells with large intercellular gaps and contained amorphous material, which was probably tissue fluid. Furthermore, this study indicated that the cells lining the lymphatic sinus-like structures put out valve-like cytoplasmic processes into the lumen. However, this study also pointed out that the ultrastructure in the outer choroid differed from typical lymphatics in the point of the discontinuous cell lining with large gaps.

 Lymphatic vessel endothelial hyaluronic acid receptor (LYVE-1) is widely accepted as the most reliable lymphatic marker that is also expressed by a subpopulation of macrophages [4, 42]. A recent paper on human choroid checked for lymphatic vessels with immunohistochemistry of LYVE-1 and podoplanin. LYVE-1(+) podoplanin(+) lymphatic vessel could not be observed and all LYVE-1(+) cells were expressed macrophage markers in human choroid [73]. These findings can support observation that the choroid contains some LYVE-1(+) macrophages and no lymphatics [74]. In corneal inflammation, LYVE-1(+) macrophages transdifferentiate and contribute to lymphangiogenesis [42]. However, the role of LYVE-1(+) macrophages has not been examined. It is unknown whether LYVE-1(+) macrophages are vital for choroidal homeostasis or how these cells conduct themselves in the pathological condition. Because the outer choroid is recognized as an unconventional route of aqueous humor outflow, choroid may have lymphatic-like system despite the lack of authentic lymphatic vessels in human.

## 15. Lymphatic-Targeting Therapy in Choroidal/Uveal Disorders

 Choroidal neovascularization (CNV), which involves abnormal growth of blood vessels in the back of the eyes, is a hallmark of age-related macular degeneration (AMD). A paper reported that both VEGF-C and VEGF-D were markedly expressed in the retinal pigment epithelium (RPE) in a surgically removed subretinal vascular membrane of AMD patients [75]. Because VEGF-C and VEGF-D show angiogenic potential, they may contribute to CNV formation. Further investigation is necessary to estimate the contribution to AMD pathogenesis (Figure 4).

 Uveitis in humans is an inflammatory and immune disease in the eye that causes severe vision loss [76]. The eye is thought to have no lymphatic drainage, and the uvea may act as an accessory lymph node during the immune response. However, the role of lymphatic system in uveitis is unclear (Figure 4). Because lymphatic system contributes to the pathogenesis of immune diseases, lymphatic-targeting drug may provide agents for uveitis treatment.

## 16. Ocular Tumor-Associated Lymphangiogenesis

 Lymphangiogenesis is observed in many types of solid tumors [77]. Human cancers express various lymphangiogenic factors including VEGF-C. Furthermore, many clinical studies showed positive correlation between VEGF-C, lymphatic invasion, lymph node metastasis, and poor patient survival. What about ocular tumors? A paper by Heindl et al. analyzed the correlation of tumor-associated lymphangiogenesis and malignancy in conjunctival squamous cell carcinoma (SCC) [52] (Table 2, Figure 4). They observed that the development of conjunctival SCC from premalignant stage was accompanied by conjunctival lymphangiogenesis. The study also noticed that the lymphangiogenesis in tumor was associated with an increased risk of local recurrence in patients with SCC. Another study evaluated whether lymphangiogenesis could contribute to the prognosis of ciliary body melanoma with extraocular extension [53]. Intraocular lymphatic vessels were found in 60% of the melanoma with extraocular extension and the lymphangiogenesis is associated with an increased mortality risk. However, uveal melanoma does not include lymphangiogenesis despite expressions of VEGF-C and its receptor VEGFR-2 and VEGFR-3 [78]. The tumor location, tumor type, or tumor malignancy can be a contributing factor to this variation. The contribution of VEGF-C to tumor progression remains unclear. Lymphangiogenesis in some ocular tumors may play an important role for the tumor progression. In the future, it will be possible that antilymphangiogenic treatment will lower metastasis rate of ocular tumor and mortality.

## 17. Ocular Drainage System

 A recent paper shows that various lymphatic markers are expressed in the human anterior segment [79]. Podoplanin is widely accepted as a reliable lymphatic marker, because of its continuous expression in lymphatic endothelium [80]. The immunohistochemical examination for podoplanin and other lymphatic markers reveals that the anterior eye segment does not have lymphatic vessels. Interestingly podoplanin can be expressed on almost all cells of the trabecular meshwork, endothelial cells of Schlemm's canal, and cells of anterior ciliary muscle tips despite the lack of lymphatics. This suggests that the aqueous humor outflow tissues have similar characteristics of lymphatic vessels (e.g., immune cell way to lymph node). Intracameral injected fluorescent antigens can be observed in the ipsilateral lymph node of the head and neck within 24 hours, suggesting that the antigen in the anterior chamber reaches the lymphoid organ [58]. This route to travel to the lymph node must be via conjunctival lymphatic and blood circulation. Furthermore, an alternative pathway via trabecular meshwork may exist. Lymphatic-related molecular or cellular targeting strategies will offer novel approaches in the treatment of inflammatory or immunological disorders in anterior ocular segments. Aqueous humor drainage from the eye is known to travel via two pathways: conventional pathway (trabecular meshwork) and alternative pathway (uveoscleral outflow) (Figure 4). Impaired aqueous humor drainage elevates intraocular pressure and results in glaucoma. A lymphatic outflow from the eye has been considered to be absent [81]. Interestingly, a third pathway was recently reported, “uveolymphatic pathway” [51] (Figure 4, Table 2). Immunogold stain as well as immunohistochemistry with podoplanin or LYVE-1 antibody showed lymphatic vessels in the human ciliary body (Figure 1). Furthermore, intracamerally injected tracer could be detected in several lymph nodes (e.g., cervical lymph node). The uveolymphatic pathway may be a novel therapeutic target for glaucoma patients.

 In 1989, McGetrick et al. searched for lymphatic drainage from monkey orbit. They injected colloid solution, or India ink, into the retrobulbar space and examined if they could reach the lymphatic vessels or the lymph node. However, no lymphatic vessels could be identified in the orbit and these tracers left the posterior orbit. This paper concluded that the posterior pathway did not lead to the lymphatic vessels or the lymph node [82]. From these observations, the human orbit has been thought to lack lymphatic vessels in a long time [83]. However, in 1993, a paper using an enzymatic method in monkey demonstrated the presence of lymphatic vessels in the orbital arachnoid, lacrimal gland, extraocular muscle, and connective tissue at the orbital apex [84] (Figure 1). In 1999, an electron microscopic study with India ink showed that human optic nerve meninges have lymphatic vessels [85]. These studies suggested new evidence that cerebrospinal fluid drained into lymphatics within the meninges of the intraorbital part of the optic nerve. However, it is unclear how lymphatics within the meninges can affect ocular disorders including glaucoma, optic neuritis, or optic neuropathy.

## 18. Conclusion

 Increasing evidence shows that lymphangiogenesis, as well as angiogenesis, has a key role in ocular physiology and pathology (Table 2). Recently antiangiogenic therapy (e.g., bevacizumab) is widely used for various ocular diseases. However, various ocular disorders including edema or inflammation still remain as a cause of visual loss. Better understanding of lymphatics and lymphangiogenesis in the eye will provide a basis for the development of novel therapeutic strategies for incurable ocular diseases (Figure 4).

## Figures and Tables

**Table 1:** Growth factors and cytokines in corneal lymphangiogenesis assay. Various growth factors or cytokines induce lymphangiogenesis as well as angiogenesis with or without VEGFR-3 activation.

Growth factor/cytokine	Angiogenesis	Lymphangiogenesis	Via VEGFR-3	Reference
VEGF-A (160–200 ng)	++	+/−	Yes/No	[22–24]
VEGF-A (400 ng)	++	+	?	[31]
VEGF-C (160–400 ng)	+	+/++	Yes	[22, 25]
VEGF-C156S (80 ng)	+	+	Yes	[26]
FGF-2 (12.5 ng)	−	+	Yes	[30]
FGF-2 (80–100 ng)	++	++	Yes	[29]
HGF (280 ng)	+	+	Yes	[28]
PDGF-BB (320 ng)	+	+	No	[27]
IGF-1 (1 *μ*g)	+	+	No	[21]
IL-1*β* (30–50 ng)	+	+	Yes	[32, 33]

**Table 2:** Lymphatic-associated ocular diseases. The list shows several diseases of the eye that are related to lymphatics or lymphangiogenesis. Further investigation might provide evidence of the contribution of lymphatics or lymphangiogenesis in the other eye diseases.

Eye diseases	Possible role of lymphatics	Reference
Corneal transplant	Lymphatic vessels but not angiogenic vessels are important for the immune rejection	[50]
Dry eye	Dry eye, which is a low-grade corneal inflammatory disorder, induces lymphangiogenesis	[49]
HSV-1 keratitis	Corneal herpes simplex virus-1 infection induces lymphangiogenesis via VEGF-A	[48]
Glaucoma	“Uveolymphatic pathway”; lymphatics exists in the ciliary body	[51]
Intraocular tumors	“Tumor-associated lymphangiogenesis” correlates the malignancy	[52, 53]
